# The nicotinamide mononucleotide adenylyl transferase (NMNAT/Nma1) modulates phosphate-sensing (*PHO*) signaling independent of its NAD^+^ synthesis activity in *Saccharomyces cerevisiae*

**DOI:** 10.1093/femsyr/foag041

**Published:** 2026-08-19

**Authors:** Yi-Ching Lee, Chi-Chun Huang, Matilda McDaniel, Su-Ju Lin

**Affiliations:** Department of Microbiology and Molecular Genetics, College of Biological Sciences, University of California, Davis, CA 95616, United States; Department of Microbiology and Molecular Genetics, College of Biological Sciences, University of California, Davis, CA 95616, United States; Department of Microbiology and Molecular Genetics, College of Biological Sciences, University of California, Davis, CA 95616, United States; Department of Microbiology and Molecular Genetics, College of Biological Sciences, University of California, Davis, CA 95616, United States

**Keywords:** NAD^+^ metabolism, phosphate sensing, nicotinamide mononucleotide adenylyl transferase NMNAT, Pho4 transcription factor, *Saccharomyces cerevisiae*, yeast molecular genetics

## Abstract

Regulation of NAD^+^ metabolism is interconnected with multiple nutrient-sensing pathways and cellular processes. The phosphate (Pi)-sensing (*PHO*) signaling pathway contributes to NAD^+^ degradation, and *PHO*-responsive genes are reciprocally regulated in a NAD^+^-dependent manner. In this study, we examine whether the NAD^+^ biosynthetic enzyme Nma1 has a direct role in modulating *PHO* signaling. We show that Nma1 physically interacts with Pho4, a transcription factor translocating to the nucleus to activate *PHO*-responsive genes during Pi depletion. Overexpression of *NMA1* or its catalytically inactive variant significantly reduces Pi depletion-induced Pho4 nuclear localization and the activation of *PHO*-responsive genes, indicating the NAD^+^ synthesis activity of Nma1 is dispensable in the downregulation of *PHO* signaling. Interestingly, mutating the C-terminal domain of Nma1, which is required for the ATPase chaperone activity of mammalian NMNATs, fails to decrease Pho4 nuclear localization and the expression of *PHO*-responsive genes. Moreover, loss of Nma1 increases Pho4 nuclear localization under moderate Pi-depleted conditions, suggesting that cells lacking Nma1 are more sensitive to Pi availability alterations. These results support that Nma1 can moderate *PHO* activation by maintaining Pho4 in the cytoplasm. Our findings uncover a novel regulatory mechanism for *PHO* signaling, and this regulation may help coordinate NAD^+^ metabolism with Pi homeostasis.

## Teaser

An NAD^+^ biosynthetic enzyme can modulate phosphate-sensing activity, linking the regulation of NAD^+^ homeostasis with nutrient signaling.

## Introduction

Nicotinamide adenine dinucleotide (NAD^+^) is an essential metabolite involved in various cellular processes. NAD^+^ and its reduced form, NADH, together mediate numerous metabolic redox reactions. NAD^+^ also serves as a substrate for NAD^+^-consuming enzymes such as sirtuins (Imai et al. 2000, Landry et al. 2000, Smith et al. 2000), poly-ADP-ribose polymerases (PARPs) (Kraus 2015), CD38 (Camacho-Pereira et al. 2016), CD157 (Ortolan et al. 2019), and SARM1 (Waller and Collins 2022). Consequently, NAD^+^ is crucial in maintaining proper cellular function, and NAD^+^ dysregulation has been implicated in many human diseases (Katsyuba et al. 2020, Xie et al. 2020, Zapata-Perez et al. 2021, Waddell et al. 2023, Lautrup et al. 2024). Nevertheless, due to the complex nature of the NAD^+^ biosynthesis pathways, the regulation of NAD^+^ homeostasis remains incompletely understood.

Biosynthesis of NAD^+^ proceeds through either *de novo* synthesis of quinolinic acid (QA) from the amino acid L-tryptophan (L-aspartate for some bacteria) or salvaging NAD^+^ intermediates, such as nicotinic acid (NA), nicotinamide (NAM), and nicotinamide riboside (NR) (Cheng and Roth 1995, Belenky et al. 2007, Lu et al. 2009, Croft et al. 2020, Groth et al. 2021). In budding yeast, the NA-NAM salvage pathway is the preferred route for NAD^+^ synthesis when NA, the major source of NAD^+^ precursors in standard yeast growth media, is abundant. NA can also be salvaged from NAM generated in NAD^+^ salvage and NAD^+^-consuming reactions by the nicotinamidase Pnc1 (Ghislain et al. 2002). NA is converted to nicotinic acid mononucleotide (NaMN) by the phosphoribosyl transferase Npt1 (Rajavel et al. 1998). The nicotinamide mononucleotide adenylyl transferases (NMNATs), Nma1 and Nma2 (Emanuelli et al. 1999), transfer the AMP moiety from ATP to convert NaMN to nicotinic acid adenine dinucleotide (NaAD), which is then converted to NAD^+^ by the NAD^+^ synthetase Qns1 (Bieganowski et al. 2003). Cells can also produce NAD^+^ through the NR salvage pathway (Belenky et al. 2007, Lu et al. 2009), in which NR is first phosphorylated by the NR kinase Nrk1 (Bieganowski and Brenner 2004), generating nicotinamide mononucleotide (NMN). NMN is then adenylated to NAD^+^ by the NMNATs Nma1, Nma2 (Emanuelli et al. 1999), and Pof1 (Kato and Lin 2014). NR salvage also connects to NA-NAM salvage because NR can be converted to NAM by the nucleosidases Urh1, Pnp1, and Meu1 (Belenky et al. 2007). On the other hand, *de novo* NAD^+^ synthesis is mediated by the Bna proteins through a series of enzymatic reactions (Panozzo et al. 2002, Wogulis et al. 2008), and after producing NaMN, the *de novo* synthesis pathway converges with the NA-NAM salvage pathway. In yeast, expression of the *BNA* genes is regulated by the NAD^+^-dependent histone deacetylase Hst1 (Bedalov et al. 2003, Groth et al. 2022) and, therefore, *de novo* synthesis activity in yeast is typically repressed until cells sense NAD^+^ depletion. However, it is unclear whether a similar NAD^+^-dependent regulation of the *de novo* pathway is conserved in other systems.

It is shown that NAD^+^ homeostasis is linked to several nutrient-sensing pathways, including phosphate (Pi)-sensing (*PHO*), amino acid-sensing Ssy1-Ptr3-Ssy5 (SPS), and cyclic AMP-dependent protein kinase A (PKA) (Lu and Lin 2011, Tsang et al. 2015, Tsang and Lin 2015). Coupling the regulation of NAD^+^ homeostasis to these pathways is advantageous as it helps cells coordinate different pathways to modulate cellular functions in response to metabolic stress. Among the numerous signaling pathways, the Pi-sensing (*PHO*) signaling pathway has a complex interplay with NAD^+^ homeostasis. First, NR salvage activity is positively correlated with the activation of *PHO* signaling, and the *PHO*-responsive genes *PHO8* and *PHM8* contribute to most of the NR production (Lu and Lin 2011, Lee et al. 2026). Second, like the *BNA* genes, it is shown that *PHO*-responsive genes are also repressed by NAD^+^-dependent Hst1 (James Theoga Raj et al. 2019, Groth et al. 2023). Thus, Hst1 coordinates *de novo* NAD^+^ metabolism and *PHO* signaling in a NAD^+^-dependent manner. In addition to Hst1-mediated regulation, *PHO*-responsive genes and *BNA* genes are activated by the transcription factor complexes Pho2-Pho4 (Lenburg and O'Shea 1996) and Pho2-Bas1 (Pinson et al. 2019), respectively. It is suggested that the competition for Pho2 between these two complexes may help modulate the balance of NAD^+^ metabolism and *PHO* activation (Groth et al. 2023). Moreover, Pho2 also appears to downregulate the NA-NAM salvage activity by repressing the expression of the nicotinamidase Pnc1 (Groth et al. 2023).

While NAD^+^ depletion can activate *PHO*-responsive genes due to the loss of Hst1 activity (Lu and Lin 2011, Groth et al. 2022, Groth et al. 2023), the level of intracellular Pi is the primary factor regulating the *PHO* signaling through modulating the localization of the transcription activator Pho4 (Auesukaree et al. 2004). When Pi is abundant, the cyclin-cyclin-dependent kinase (CDK) complex, the Pho80-Pho85 complex, phosphorylates the transcription factor Pho4 (Kaffman et al. 1994). Phosphorylated Pho4 is exported from the nucleus (Kaffman et al. 1998b), and the *PHO*-responsive genes remain inactivated. When Pi is scarce, the Pho80-Pho85 complex is inhibited by the CDK inhibitor Pho81 (Schneider et al. 1994). Moreover, the levels of inositol heptakisphosphate (IP_7_) increase upon Pi depletion (Auesukaree et al. 2005, Lee et al. 2007). IP_7_ modulates the interaction between Pho81 and Pho80-Pho85 complex, which blocks the active site of Pho85 and prevents Pho4 phosphorylation (Lee et al. 2008). Unphosphorylated Pho4 accumulates in the nucleus and forms a complex with Pho2 (Vogel et al. 1989, Barbaric et al. 1998) to activate the *PHO*-responsive genes (Ogawa et al. 2000). Overall, the phosphorylation of Pho4 determines Pho4 localization and the activation of *PHO* signaling (Komeili and O'Shea 1999). On the other hand, *PHO* signaling can also be activated by the purine metabolite ZMP (5-amino-4-imidazole carboxamide ribonucleotide 5’-phosphate; AICAR monophosphate), which facilitates Pho2-Pho4 physical interaction (Pinson et al. 2009). It is suggested that (S)ZMP, which include ZMP and its precursor succinyl-ZMP, serve as signals for purine and ATP shortage (Pinson et al. 2019). Since Pi is a component for nucleotide synthesis, it is conceivable that yeast cells utilize the purine metabolites (S)ZMP to maintain purine-Pi homeostasis by co-regulating these two metabolic pathways (Pinson et al. 2009). NAD^+^ metabolism has also been connected to (S)ZMP signaling through co-regulation of *de novo* purine synthesis and *de novo* NAD^+^ synthesis by the Pho2-Bas1 complex (Pinson et al. 2019). Moreover, one of the NAD^+^ intermediates, NAM, may co-regulate *PHO* signaling and *de novo* NAD^+^ synthesis by modulating Hst1 activity (James Theoga Raj et al. 2019). Whether additional NAD^+^-associated metabolic factors also play a role in the co-regulation of NAD^+^ metabolism and *PHO* signaling remains to be investigated.

NNMATs are NAD^+^ biosynthetic enzymes involved in both the *de novo* synthesis and salvage pathways. In budding yeast, Nma1 is the rate-limiting enzyme for NAD^+^ synthesis. Overexpression of *NMA1* effectively raises cellular NAD^+^ levels, whereas deletion of *NMA1* significantly reduces NAD^+^ levels (Croft et al. 2018, Pinson et al. 2019, Lee et al. 2026). Interestingly, overexpression of *NMA1* appears to protect cells from proteotoxicity (Ocampo et al. 2013) independent of its NAD^+^ synthesis activity (Pinkerton et al. 2021). In line with this, studies have shown that NMNATs are multifunctional proteins with chaperone-like activity, alleviating neurodegeneration in fly (Zhai et al. 2006, Zhai et al. 2008) and mouse models (Ma et al. 2020). These studies overall suggest that NMNATs are not merely involved in NAD^+^ production. It has been reported in a proteomics study that Nma1 physically interacts with Pho4 (Graumann et al. 2004), suggesting a role for Nma1 in the regulation of the *PHO* signaling pathway. In this study, we examined the impacts of Nma1 on Pho4 localization and *PHO* signaling. Our studies help understand the role of Nma1 in the interconnection between NAD^+^ metabolism and Pi homeostasis.

## Materials and methods

### Yeast strains, growth media, and plasmids

Yeast strain BY4742 *MATα his3Δ1 leu2Δ0 lys2Δ0 ura3Δ0* acquired from Open Biosystems (Brachmann et al. 1998) was used as the WT strain for this study. Synthetic Dextrose (SD) media were made as described (Burke et al. 2000). All standard growth media are phosphate-replete (normal, +Pi). Phosphate-depleted (-Pi) media were prepared by Pi precipitation as previously described (Lu and Lin 2011). In brief, 2.46 g of MgSO_4_ was first dissolved in one liter of media, and then 8 mL of concentrated ammonia was slowly added with gentle stirring to precipitate inorganic Pi as MgNH_4_PO_4_. After filtration, HCl was added to adjust pH to 6, followed by autoclave sterilization. For Pi depletion studies, cells were first grown to early logarithmic phase in regular media, washed twice with Pi-depleted media, and then switched to Pi-depleted media for the indicated time points ranging from 30 min to 2 h. Gene deletion was performed using the standard PCR-based strategy to amplify gene-specific PCR products generated using either the reusable loxP-kanMX-loxP (pUG6) (Guldener et al. 1996) or the pAG32-hphMX4 (Goldstein and McCusker 1999) cassettes as templates. The HA or Myc or EGFP epitope tag was added to the C-terminus of target genes in the genome using the pFA6a-3HA-HIS3MX (Longtine et al. 1998) or pFA6a-13MYC-KANMX (Longtine et al. 1998) or pFA6a-link-yoEGFP-CaURA3 (Sheff and Thorn 2004, Lee et al. 2013) (Addgene Cat# 44 872) plasmid as the template for PCR-mediated tagging. These C-terminally tagged proteins were used for protein expression studies and their expression are driven by their native promoter. For co-immunoprecipitation, the HA tag was added to the N-terminus of *PHO4* in the genome using the pFA6a-kanMX6-PGAL1-3HA plasmid as the template for PCR-mediated tagging (Longtine et al. 1998). The expression of N-terminally tagged Pho4 is driven by *GAL1* promoter in the absence of *GAL80* to increase Pho4 expression in regular glucose media. The *NMA1* overexpression construct was made in the integrative pPP81 (*LEU2*) vector as described (Easlon et al. 2008), and gene overexpression is driven by the *ADH1* promoter. The coding region of *NMA1* was PCR amplified with NotI site added to the 5’ end and NheI site added to the 3’ end, which was then cloned into pPP81 digested with NotI and NheI. After PacI digestion, the linearized plasmid was integrated into the yeast genome as described (Easlon et al. 2008). Variants of Nma1 were generated using the Q5® site-directed mutagenesis kit (New England Biolabs, Cat# 0552S). Oligos used for mutagenesis are listed as follows: NMA1 (H181A)-F, caatcacctacttggctctaagaatgtttg; NMA1 (H181A)-R, gtgaaaaagacccacatgc tactattac; NMA1 (R359A)-F, ttccacgaaagttgctctatttatcagacgc; NMA1 (R359A)-R, gaaatatcattatagatgagttgcttgatgataagaatattcc; NMA1 (ΔCT)-F, caagttcttggaaacaaagaatg; NMA1 (ΔCT)-R, attaggtaacaaatattgtacagacatg. All plasmid constructs were verified by DNA sequencing.

### Co-immunoprecipitation (Co-IP) analysis

Cells were grown in SD for 5–6 h at a starting OD_600_ = 0.2 to a density of OD_600_ = 1.0, and 50 OD_600_ unit cells were collected by centrifugation. For Pi depletion, cells were grown in regular SD for 4 h at a starting OD_600_ = 0.2 to a density of OD_600_ = 0.6–0.8. Then cells were washed twice with Pi-depleted SD and cultured in Pi-depleted SD for 2 h before collecting 50 OD_600_ unit cells. Collected cells were snap-frozen in liquid nitrogen before extraction. Protein extracts were obtained by bead-beating (Biospec Products) in 500 µl lysis buffer: 50 mM Na–HEPES, pH 7.5, 200 mM NaOAc, pH 7.5, 1 mM EDTA (pH = 8), 1 mM EGTA, 5% glycerol, 0.25% NP-40, 3 mM DTT, 1 mM PMSF, and protease inhibitor cocktail (Pierce, Cat# A32955). The whole cell lysates were drawn off the beads, moved to new tubes, and then centrifuged at a maximum speed (13 200 rpm) for 20 min at 4°C. A 20 µl sample of total cell lysate was set aside and mixed with an equal volume of 2x loading buffer (Input). About 500 μl of the whole cell lysates (∼10 mg/ml) were then mixed with 50 μl anti-HA magnetic beads (Pierce, Cat# 88 836) and incubated overnight at 4°C with gentle agitation. The beads were washed three times with 1 ml 0.05% TBST at room temperature to remove unbound proteins. The IP samples were eluted from the beads using 50 μl SDS sample buffer and incubated at 100°C for 10 min. The collected IP samples (10 μl) and input samples (10 μl, 100x dilution for Nma1) were then subjected to immunoblot analysis. To verify that the wash conditions effectively removed non-specific binding, we blotted for Pgk1 as a negative control in the first set of experiments. As expected, Pgk1 was detected in the input fraction, but not in the IP fraction (data not shown).

### Protein extraction and immunoblot analysis

To study Pho4 protein expression, cells were grown in SD for 5–6 h at a starting OD_600_ = 0.2 to a density of OD_600_ = 1.0, and 15 OD_600_ unit cells were collected by centrifugation. For Pi depletion, cells were grown in SD for 4 h at a starting OD_600_ = 0.2 to a density of OD_600_ = 0.6–0.8. Cells were then washed twice with Pi-depleted SD and cultured in Pi-depleted SD for 2 h before collecting 15 OD_600_ unit cells. Collected cells were snap-frozen in liquid nitrogen before extraction. Protein extracts were obtained by bead-beating (Biospec Products) in lysis buffer: 50 mM Tris–HCl, pH 7.5, 100 mM NaCl, 1% Triton X-100, 5 mM EDTA (pH = 8), 1 mM PMSF, and protease inhibitor cocktail (Pierce, Cat# A32955). Protein concentrations were estimated using the Bradford assay reagent (Bio-Rad, Cat# 5000006), comparing against a BSA standard curve. 10 μg (Pgk1) or 30 μg (Pho4) of total protein was loaded in each lane. After electrophoresis, the protein was transferred to a polyvinylidene fluoride membrane (Cytiva Amersham, Cat# 45004021). Blocking was carried out using OneBlock Western-CL Blocking buffer (Genesee, Cat# 20313) or EveryBlot Blocking Buffer (Bio-Rad, Cat# 12010020) at room temperature for 1 h. The membranes were then washed and blotted with either rabbit anti-HA antibody (Cell Signaling, Cat# 3724S, 1:2000 dilution), mouse anti-PGK antibody (Invitrogen, Cat# 459250, 1:5000 dilution), or mouse anti-Myc antibody (Sigma, Cat# 05724, 1:2000 dilution) for overnight incubation at 4°C. Protein was visualized using anti-mouse or anti-rabbit immunoglobulin antibody conjugated to horseradish peroxidase (Invitrogen, Cat #31430 and #31460, 1:10 000 dilution) and the ECL reagents (Cytiva Amersham, Cat# 45010090). For each protein, multiple images were taken with multiple exposure times (10 s increments) to avoid signal saturation. The chemiluminescent image was analyzed using the Amersham Imager 600 (GE) system and software provided by the manufacturer.

### Fluorescence microscopy

Cells were first grown in SD without uracil (to select for *URA3*-integrated GFP fusions) at a starting OD_600_ = 0.2 to a density of OD_600_ = 0.8–1.0 before Pi depletion. After Pi depletion, 4 OD_600_ unit cells were collected by centrifugation and then resuspended in 150 μl of Pi-depleted SD without uracil. Total of 2 μl of the cell suspension was placed on the microscope slide for imaging. DAPI (Sigma, Cat# 268298, 1:1000 dilution) was added to the cell culture to label the DNA in the nucleus 2 h before imaging. Localization analysis was performed by fluorescence microscopy with a Nikon Eclipse 80i microscope at × 1000 magnification. Images were captured using an ORCA-Fusion Gen-III sCMOS CAMERA (Hamamatsu Photonics) and the HCImage software (Universal Imaging, Downingtown, Pennsylvania, United States). To enhance the fluorescence signal and lower the noise, the “Ultra Quiet” mode was selected in the capture-trigger modes-speed setting of the HCImage software. All fluorescent images were exposed for 2 s. The nuclear/cytoplasmic (N/C) Pho4-EGFP ratio was calculated as described (Kelley and Paschal 2019). In brief, the average of the fluorescence intensity was quantified by separately selecting the entire nucleus (N) or the entire cytoplasm (C) of each cell using ImageJ. The background signal was then subtracted from the average of the fluorescence intensity. The statistical analysis was performed using the GraphPad Prism 10 software. A total of 100 or 150 cells from at least three independent experiments were analyzed per strain and/or per experimental condition.

### Measurements of NAD^+^ and NADH

Cellular levels of NAD^+^ and NADH were determined using enzymatic cycling reactions as described (Easlon et al. 2008). Cells were grown in SD at a starting OD_600_ = 0.2 to a density of OD_600_ = 1.0, and 1 OD_600_ unit cells were collected in duplicate by centrifugation. Acid extraction was used to obtain NAD^+^, and alkali extraction was used to obtain NADH at 60°C for 40 min. Amplification of NAD^+^ or NADH in the form of malate was carried out using 3 μl (for NAD^+^) or 5 μl (for NADH) of neutralized acid- or alkali-extracted lysate in 100 μl of cycling reaction at room temperature for 1 h. The reaction was terminated by heating at 100°C for 5 min. Next, malate produced from the cycling reaction was converted to oxaloacetate and then to aspartate and α-ketoglutarate by the addition of 1 ml malate indicator reagent at room temperature for 20 min. The reaction produced a corresponding amount of NADH as readout, which was measured fluorometrically with excitation at 365 nm and emission monitored at 460 nm (GloMax, Promega). Standard curves for determining NAD^+^ and NADH concentrations were obtained as follows: NAD^+^ and NADH were added to the acid and alkali buffer to a final concentration of 0, 2.5, and 7.5 μM, which were then treated with the same procedure along with other samples. The fluorometer was calibrated each time before use with 0, 5, 10, 20, 30, and 40 μM NADH to ensure that the detection was within a linear range.

### Quantitative PCR (qPCR) analysis of gene expression levels

Cells were grown in SD for 5–6 h at a starting OD_600_ = 0.2 to a density of OD600 = 1.0, and 40 OD_600_ unit cells were collected by centrifugation. For Pi depletion, cells were first grown in SD for 5 h at a starting OD_600_ = 0.2 to a density of OD_600_ = 0.8. Cells were then centrifuged, washed twice with Pi-depleted SD, and cultured in Pi-depleted SD for 1 h before collecting 40 OD_600_ unit cells. Total RNA was extracted using GeneJET RNA purification kit (Thermo Fisher Scientific, Cat# FERK0731), and complementary DNA was synthesized using QuantiTect Reverse Transcription kit (Qiagen, Cat# Q205313) according to the manufacturer’s instructions. About 50 ng of cDNA and 500 nM of specific primer sets were used for each qPCR reaction, which was run in 96-well plates on Roche LightCycler 480 using LightCycler 480 SYBR green I Master Mix (Roche, Cat#507203180) as previously described (Lee et al. 2026). The average size of the amplicon for each gene was approximately 150 bp. The target mRNA transcript levels were normalized to the internal control *TAF10* transcript levels.

### Statistical analysis

At least three samples (biological replicates or technical replicates) prepared from each strain per condition were used for statistical analysis to ensure adequate power for all studies. Statistical analysis was carried out by ordinary one-way *ANOVA* or two-way *ANOVA* with multiple testing correlation within multiple groups or two-tailed Student’s *t* test within two groups using GraphPad Prism 10. Data were presented as mean ± SD. *P* < 0.05 is considered significant.

## Results

### Nma1 physically interacts with Pho4 independent of phosphate availability

**Figure 1:**
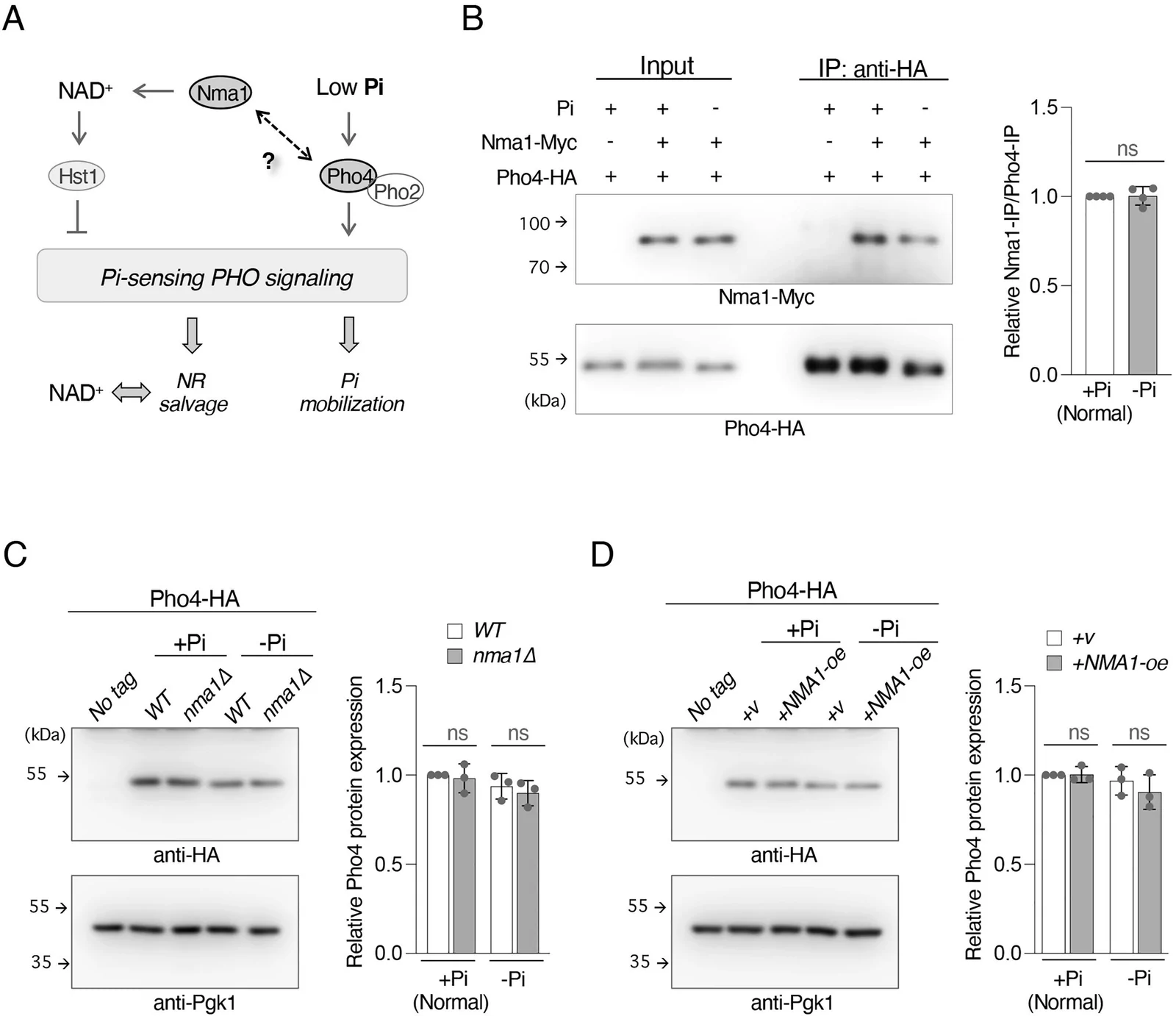
(A) An illustration of the interplay between NAD^+^ and *PHO* signaling. Nma1 plays a role in NAD^+^ synthesis and possibly affects *PHO* signaling via Pho4. (B) Co-immunoprecipitation showing that Nma1 physically interacts with Pho4 regardless of Pi availability. Yeast cell extracts were immunoprecipitated with an anti-HA antibody, followed by immunoblotting with the indicated antibodies. Input sample represents about 1% of the total lysate used for immunoprecipitation; the IP sample represents about 20% of the immunoprecipitated proteins. More details are shown in the Materials and methods. The image is representative of the trend observed across four independent experiments (left panel). Relative Nma1 IP level is normalized to the Pho4 level in the IP fraction, with the level of the normal condition (+Pi) set to 1 (right panel). Error bars represent data from four biological replicates. (C) Comparison of Pho4 protein expression by Western blot analysis. Results show that deletion of *NMA1* does not affect Pho4 protein levels in both normal (+Pi) and Pi-depleted (-Pi) conditions. (D) Overexpression of *NMA1* does not affect Pho4 protein levels in both normal and Pi-depleted conditions. For (C) and (D), relative protein expression is normalized to Pgk1, and the protein expression of Pho4-HA in WT cells in normal condition is set to 1 (right panel). Error bars represent data from three biological replicates. The *P* values are calculated using Student’s *t* test. (*, *P* < 0.05; **, *P* < 0.005; ***, *P* < 0.0005; ****, *P* < 0.0001; ns, not significant)

To further study the interplay between NAD^+^ metabolism and *PHO* signaling (Fig. 1A), we first examined the potential interaction between the NAD^+^ biosynthetic enzyme Nma1 and the transcription factor Pho4. A previous study, which employed the multidimensional protein identification technology (MudPIT) to investigate protein interactions targeting proteins involved in transcription and mitosis, has reported that Pho4 physically interacts with Nma1 (Graumann et al. 2004). To validate this, we performed co-immunoprecipitation (Co-IP) analysis using the HA-tagged Pho4 and the Myc-tagged Nma1. To further examine whether Pi availability would affect the binding efficiency between Nma1 and Pho4, we carried out the studies in both normal (+Pi) and Pi-depleted (-Pi) conditions. As shown in Fig. 1B, the Myc-tagged Nma1 was observed in the IP fractions, indicating that Pho4 indeed has a physical interaction with Nma1. When we further compared the relative Nma1 levels in the IP fractions (normalized to Pho4 levels in the same IP fractions), no significant difference was found between the normal and Pi-depleted conditions (Fig. 1B, right), suggesting that Nma1 binds to Pho4 regardless of Pi availability. Moreover, studies have shown that NMNAT is a dual-function protein that acts as both a NAD^+^ biosynthetic enzyme and a molecular chaperone (Zhai et al. 2008, Brazill et al. 2017, Ma et al. 2020). Therefore, it is possible that when Nma1 interacts with Pho4, the chaperone activity of Nma1 may affect Pho4 protein level or activity. To address this, we determined Pho4 protein expression in both normal and Pi-depleted conditions. However, Pho4 expression was not significantly altered in either *nma1Δ* (Fig. 1C) or *NMA1*-oe cells (Fig. 1D).

### Overexpression of Nma1 limits Pho4 nuclear localization independent of its NAD^+^ synthesis activity

**Figure 2:**
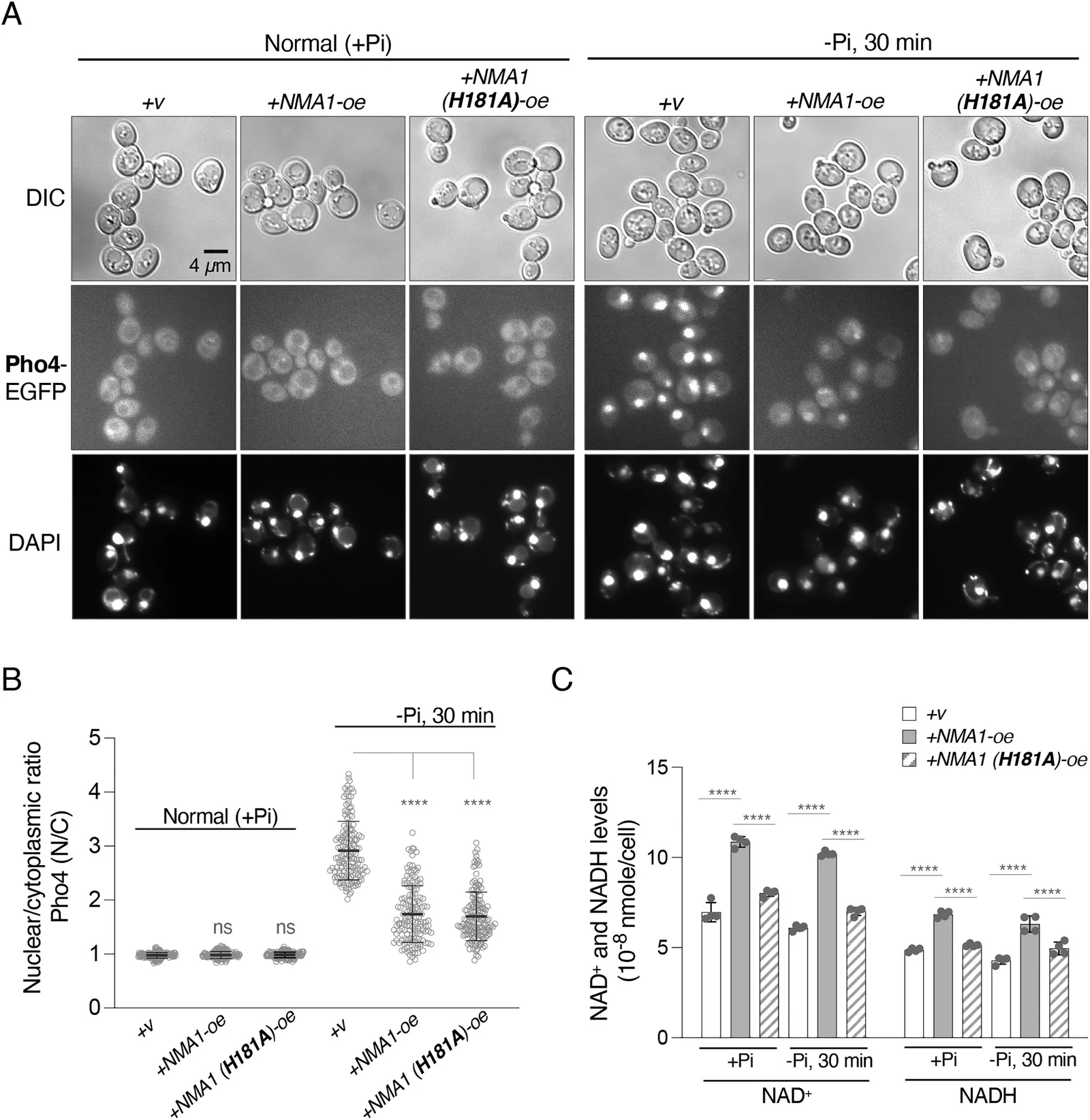
(A) Fluorescence microscopy showing that overexpression of the WT *NMA1* or the NAD^+^ synthesis-deficient *NMA1* (H181A) reduces Pho4 nuclear localization in Pi-depleted (-Pi) conditions. To observe Pho4 translocation, cells were first cultured in regular SD (+Pi) without uracil (to select for *URA3*-integrated GFP fusions) and then switched to Pi-depleted (-Pi) SD without uracil for 30 min before imaging. (B) Quantitative analysis of the results from (A). 150 cells per strain per condition were used to calculate the average and the error bar of the nuclear/cytoplasmic (N/C) ratio. (C) Comparisons of the NAD^+^ and NADH levels. Overexpression of the WT *NMA1* significantly increases NAD^+^(H) levels in both normal (+Pi) and Pi-depleted (-Pi) conditions, whereas the NAD^+^ synthesis-deficient Nma1 (H181A) fails to boost NAD^+^(H) levels. The graph is representative of the trend observed across three independent experiments (representing six biological replicates). Error bars represent data from two biological replicates, each with two technical replicates. For (B) and (C), the *P* values are calculated using one-way and two-way *ANOVA*, respectively. (*, *P* < 0.05; **, *P* < 0.005; ***, *P* < 0.0005; ****, *P* < 0.0001; ns, not significant)

Next, we examined whether Nma1 affects the subcellular localization of Pho4. It is shown that Pi availability regulates Pho4 localization. In Pi-rich medium, Pho4 is phosphorylated by the Pho80-Pho85 complex, which results in cytoplasmic localization (Kaffman et al. 1994, O’Neill et al. 1996). Conversely, Pho4 is concentrated in the nucleus when cells are starved for Pi (O’Neill et al. 1996). To observe Pho4 localization, we tagged Pho4 with the enhanced green fluorescent protein (EGFP) (Sheff and Thorn 2004, Lee et al. 2013) and observed its localization using fluorescence microscopy. Consistent with previous reports (Kaffman et al. 1994, O’Neill et al. 1996), Pho4 displayed a cytoplasmic localization when cells were grown in Pi-replete regular medium, whereas Pho4 was enriched in the nucleus when cells were grown in Pi-depleted medium (Fig. 2A). Interestingly, overexpression of *NMA1* appeared to decrease Pho4 nuclear localization upon Pi depletion (Fig. 2A). To evaluate the effect of *NMA1*-oe, we quantified the EGFP fluorescence signal in the nucleus (N) and in the cytoplasm (C) to obtain the N/C ratio, which reflects the relative nuclear abundance of Pho4 (Kelley and Paschal 2019). As shown in Fig. 2B, *NMA1*-oe significantly reduced the nuclear abundance of Pho4 in Pi-depleted condition.

As a major NAD^+^ biosynthetic enzyme, overexpression of *NMA1* effectively boosts the cellular NAD^+^ levels (Fig. 2C) (Croft et al. 2018, Pinson et al. 2019, Lee et al. 2026). It has been shown that NAD^+^ can impact *PHO* activity (Lu and Lin 2011), likely through Hst1-mediated repression of *PHO*-responsive genes (Fig. 1A) (James Theoga Raj et al. 2019, Groth et al. 2023). We therefore further investigated whether the increased NAD^+^ levels may play a role in the reduction of Pho4 nuclear localization in *NMA1*-oe cells. To address this, we mutated the NAD^+^ synthesis catalytic domain of Nma1, generating the Nma1 (H181A) mutant (Mack et al. 2001, Zhai et al. 2006, Pinkerton et al. 2021), and examined whether this NAD^+^ synthesis-deficient Nma1 could still impede Pho4 nuclear localization. We first confirmed that the NAD^+^ levels in the mutant *NMA1* (H181A)-oe cells were significantly lower than the levels in the wild-type *NMA1* (WT)-oe cells under both normal and Pi-depleted conditions (Fig. 2C). Next, we showed that overexpressing the NAD^+^ synthesis-deficient *NMA1* (H181A) mutant could still reduce Pho4 nuclear localization in cells grown in Pi-depleted medium (Fig. 2A and B), and the effect was comparable to overexpressing the wild-type *NMA1*. Together, our results suggest that Nma1 decreases Pho4 nuclear localization during Pi depletion, which is independent of its NAD^+^ synthesis activity.

### The C-terminal domain of Nma1, including an ATP-binding site, is crucial for modulating Pho4 localization

**Figure 3:**
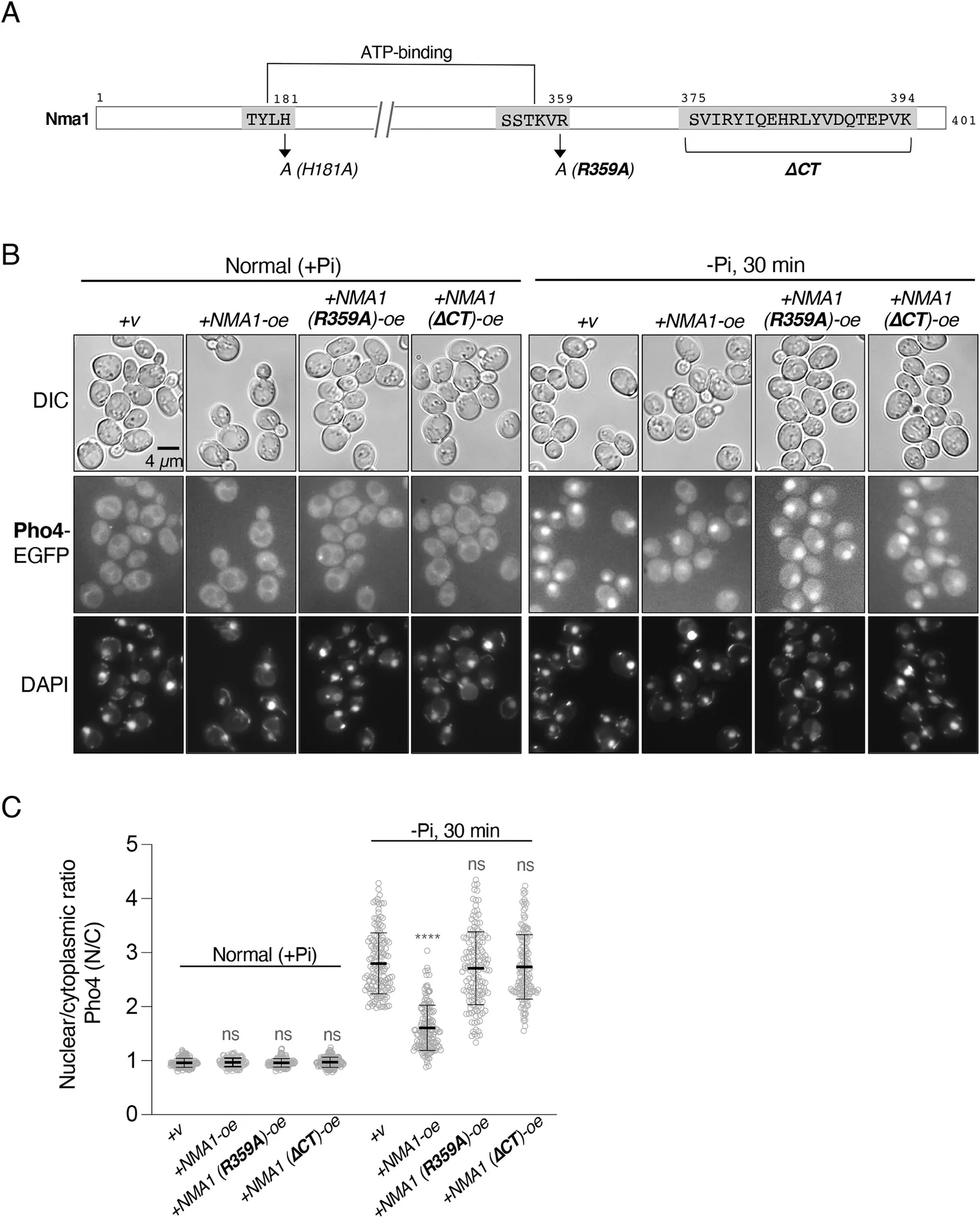
(A) An illustration showing the two ATP-binding sites of Nma1 and the mutations of the Nma1 variants used in this study. (B) Overexpression of the C-terminal variants of *NMA1* (R359A or ΔCT) does not reduce Pho4 nuclear localization in Pi-depleted (-Pi) condition. To observe Pho4 translocation, cells were first cultured in regular SD (+Pi) without uracil and then switched to Pi-depleted SD (-Pi) without uracil for 30 min before imaging. (C) Quantitative analysis of the results from (B). 150 cells per strain per condition were analyzed to calculate the average and the error bar of the nuclear/cytoplasmic (N/C) ratio. The *P* values are calculated using one-way *ANOVA*. (*, *P* < 0.05; **, *P* < 0.005; ***, *P* < 0.0005; ****, *P* < 0.0001; ns, not significant)

Next, we examined whether the putative chaperone/ATPase domain of Nma1 plays a role in modulating Pho4 nuclear localization. All NMNATs contain two conserved functional motifs, the N-terminal ATP-binding site and the C-terminal ATP-binding site (Garavaglia et al. 2002) (Fig. 3A). The N-terminal ATP-binding site is characterized as the NAD^+^ synthesis catalytic domain (Saridakis and Pai 2003, Yalowitz et al. 2004, Zhai et al. 2006), and our results showed that the N-terminal ATP-binding site of Nma1 is dispensable for modulating Pho4 nuclear localization during Pi depletion (Fig. 2A and B). Contrary to the N-terminal ATP-binding site, the C-terminal ATP-binding site is not required for NAD^+^ synthesis but correlates with chaperone activity (Ali et al. 2016). To examine whether the C-terminal ATP-binding site plays a role in modulating Pho4 localization, we mutated the conserved arginine residue in the C-terminal ATP-binding site of Nma1, generating Nma1 (R359A) (Zhai et al. 2006, 2008) (Fig. 3A). As shown in Fig. 3B, overexpressing the mutant *NMA1* (R359A) failed to reduce Pho4 nuclear abundance in Pi-depleted condition, suggesting that the C-terminal ATP-binding site of Nma1 is involved in the reduction of Pho4 nuclear localization. Supporting this, overexpressing another variant of *NMA1* lacking the entire C-terminal ATP-binding site (∆354–359) also failed to reduce Pho4 nuclear abundance in Pi-depleted condition (data not shown). Although the C-terminal ATP-binding site of Nma1 has not been directly studied for its impact on the chaperone/ATPase activity, it is shown that deletion of the further downstream C-terminal tail of Nma1 (∆CT, ∆375–394) (Fig. 3A) attenuates its chaperone activity without impacting the NAD^+^ synthesis activity (Pinkerton et al. 2021). We therefore further examined whether the C-terminal tail of Nma1 also plays a role in modulating Pho4 localization. Interestingly, overexpressing the truncated *NMA1* (ΔCT) indeed failed to decrease Pho4 nuclear abundance in Pi-depleted condition, and the effect was comparable to overexpressing the ATP-binding mutant *NMA1* (R359A) (Fig. 3B and C). These results suggest that the C-terminal ATP-binding site of Nma1 is required for the reduction of Pho4 nuclear localization in response to Pi depletion. Moreover, the further downstream C-terminal tail of Nma1 is also involved in modulating Pho4 localization, likely by facilitating ATP binding to modulate the chaperone/ATPase activity of Nma1. Overall, our data suggest that Nma1 may function as a chaperone to help maintain Pho4 in the cytoplasm.

### Deletion of *NMA1* increases Pho4 nuclear localization even in moderate phosphate-depletion conditions

**Figure 4:**
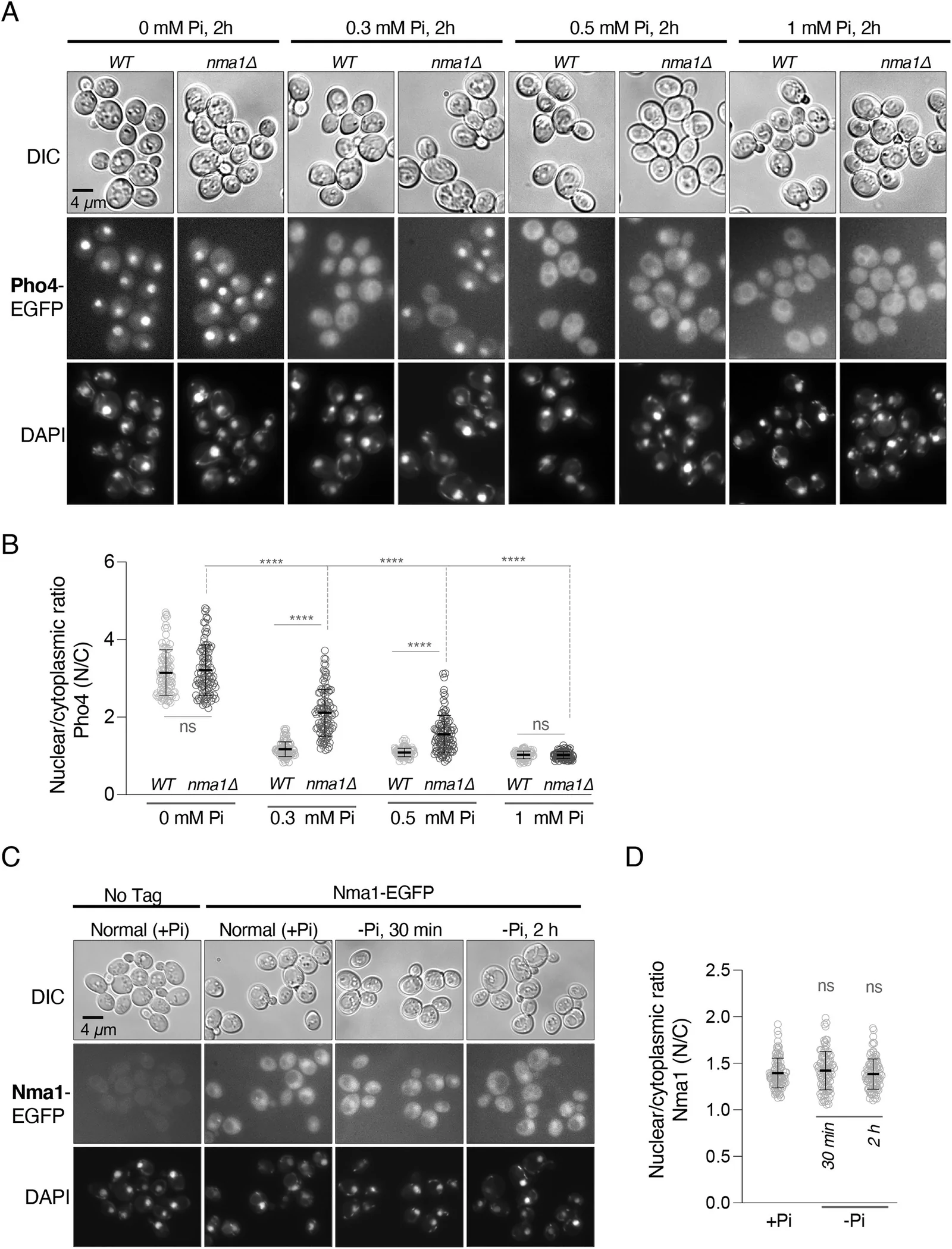
(A) Pho4 nuclear localization is increased in *nma1Δ* cells cultured in media with limited Pi (0.3 and 0.5 mM) compared to WT cells. To observe Pho4 translocation, cells were first cultured in regular SD (+Pi) without uracil and then switched to media containing different Pi concentrations for 2 h before imaging. (B) Quantitative analysis of the results from (A). 100 cells per strain per condition were used to calculate the average and the error bar of the nuclear/cytoplasmic (N/C) ratio. (C) Nma1 localization is not altered by Pi depletion. (D) Quantitative analysis of the results from (C). 100 cells per strain per condition were used to calculate the average and the error bar of the N/C ratio. For (B) and (D), the *P* values are calculated using one-way *ANOVA*. (*, *P* < 0.05; **, *P* < 0.005; ***, *P* < 0.0005; ****, *P* < 0.0001; ns, not significant)

To further strengthen our findings, we examined whether deleting *NMA1* would promote Pho4 nuclear localization. In Pi-depleted medium, Pho4 was predominantly localized in the nucleus in both WT and *nma1Δ* cells (Fig. 4A, left), which makes it difficult to evaluate the effect of *NMA1* deletion on Pho4 nuclear abundance in Pi-depleted condition. It has been shown that Pho4 localization can be modulated by moderate Pi depletion (Springer et al. 2003). For instance, Pho4 resides in the nucleus when cells are grown in medium containing 0.1 mM Pi, whereas Pho4 becomes cytoplasmic in cells cultured in medium containing 0.3 mM Pi (Springer et al. 2003). We therefore cultured cells in media supplemented with different levels of Pi (0, 0.3, 0.5, 1 mM) and observed Pho4 localization. Consistent with previous findings (Springer et al. 2003), Pho4 displayed a cytoplasmic localization in WT cells grown in media that contained 0.3 mM Pi or higher Pi concentrations (Fig. 4A). Deletion of *NMA1* significantly enhanced Pho4 nuclear abundance when cells were cultured in either 0.3 mM Pi medium or 0.5 mM Pi medium (Fig. 4A and B). In 1 mM Pi medium where Pi availability was relatively abundant, deletion of *NMA1* had no impact on Pho4 localization, and Pho4 was cytoplasmic in both WT and *nma1Δ* cells (Fig. 4A, right). To further support the model that Nma1 may maintain Pho4 in the cytoplasm, we examined the subcellular localization of Nma1 in both normal and Pi-depleted conditions. Consistent with the previous finding (Huh et al. 2003), Nma1 was distributed in both the nucleus and cytoplasm when cells were grown in regular medium (Fig. 4C). Pi depletion did not alter the localization pattern of Nma1 (Fig. 4C and D). Overall, these results suggest that loss of Nma1 renders a higher sensitivity to Pi depletion because Pho4 nuclear abundance is enhanced by moderate Pi depletion in *nma1∆* cells but not in WT cells.

### Overexpression of *NMA1* suppresses the activation of *PHO*-responsive genes

**Figure 5:**
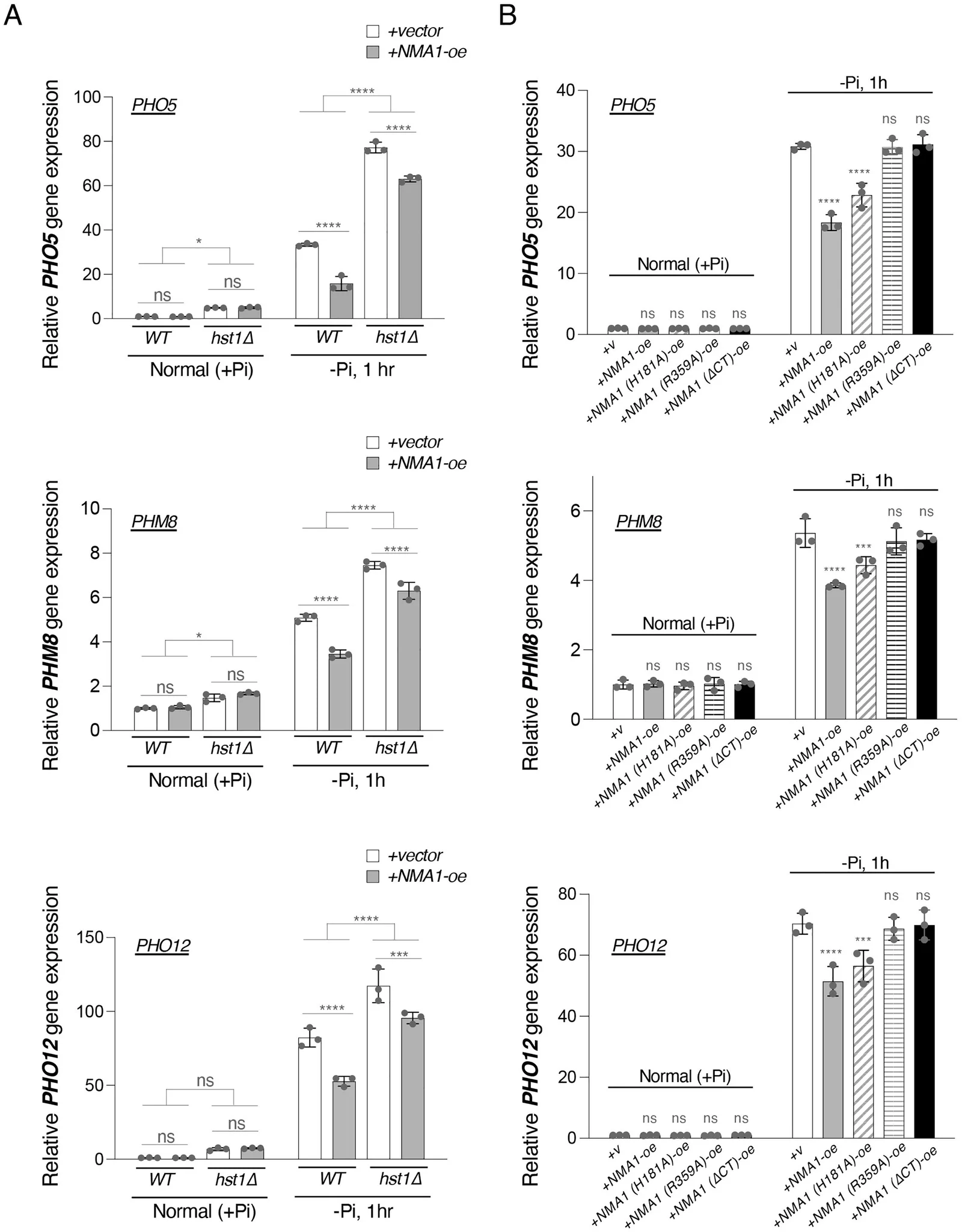
(A) Gene expression analysis of *PHO*-responsive *PHO5, PHM8*, and *PHO12* genes determined by qPCR. Overexpression of *NMA1* reduces the expression of *PHO*-responsive genes in both WT and *hst1Δ* cells in response to Pi depletion. (B) Overexpression of the WT *NMA1* and the NAD^+^ synthesis-deficient *NMA1* (H181A) reduces the expression of *PHO*-responsive genes in response to Pi depletion. To induce the activation of *PHO*-responsive genes, cells were first cultured in regular SD (+Pi) and then switched to Pi-depleted SD (-Pi) for 1 h. The graphs are representative of the trend observed across three independent experiments representing three biological replicates. Relative gene expression is normalized to *TAF10* and the gene expression of WT cells in regular SD is set to 1. Error bars represent data from three technical replicates. The *P* values are calculated using two-way *ANOVA*. (*, *P* < 0.05; **, *P* < 0.005; ***, *P* < 0.0005; ****, *P* < 0.0001; ns, not significant)

Because *NMA1*-oe reduces Pho4 nuclear localization in Pi-depleted condition (Figs. 2 and 3), we expect *NMA1*-oe would also reduce the expression of *PHO*-responsive genes. Supporting this, the expression of *PHO*-responsive genes, including *PHO5, PHO12*, and *PHM8*, was indeed significantly decreased in *NMA1*-oe cells cultured in Pi-depleted medium (Fig. 5A). On the other hand, it is reported that Hst1, an NAD^+^-dependent histone deacetylase, represses the expression of *PHO*-responsive genes (James Theoga Raj et al. 2019, Groth et al. 2023) (Fig. 1A). Therefore, it is possible that *NMA1*-oe may enhance Hst1 activity through increasing the NAD^+^ level, which may result in the reduced expression of *PHO*-responsive genes. To further confirm that *NMA1*-oe can downregulate *PHO*-responsive genes by maintaining Pho4 in the cytoplasm, we employed the *hst1Δ* cells to rule out the NAD^+^ effect. In the absence of *HST1*, the *PHO*-responsive genes were overall upregulated in both normal and Pi-depleted conditions (Fig. 5A). *NMA1*-oe was able to lower the expression of *PHO*-responsive genes in *hst1Δ* cells cultured under Pi-depleted condition (Fig. 5A), suggesting that Nma1 can regulate *PHO*-responsive genes independent of its NAD^+^ synthesis activity. In line with this, overexpressing the NAD^+^ synthesis-deficient *NMA1* (H181A) mutant also significantly lowered the expression of *PHO*-responsive genes in WT cells cultured in Pi-depleted medium (Fig. 5B). On the other hand, overexpressing the C-terminal *NMA1* mutants failed to repress the *PHO*-responsive genes (Fig. 5B). Together, these results suggest that Nma1 plays a role in modulating Pho4-mediated activation of *PHO*-responsive genes, primarily by limiting Pho4 nuclear abundance through its chaperone/ATPase activity.

## Discussion

**Figure 6:**
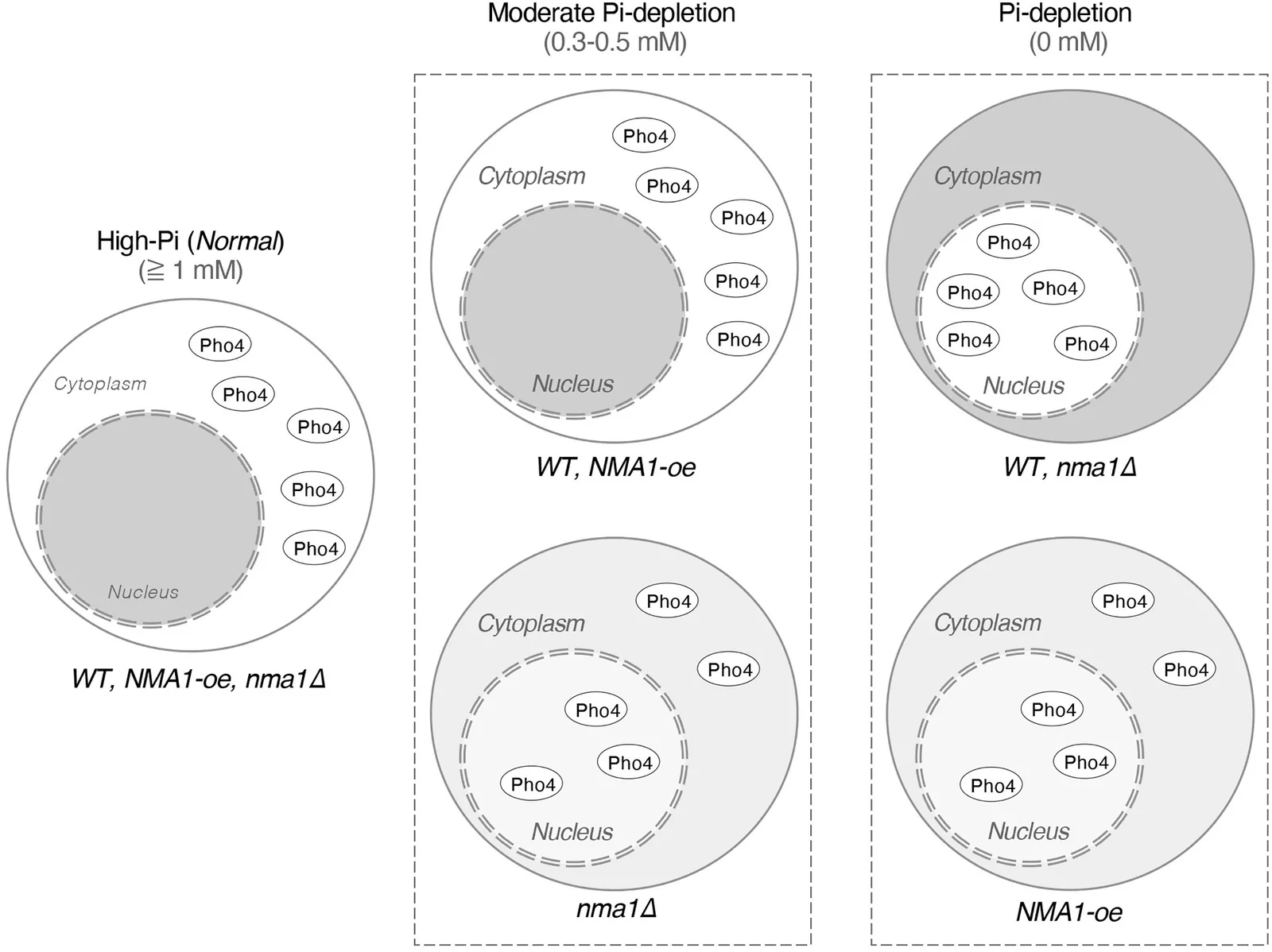
Summary model of Pho4 relative abundance and localization in response to phosphate fluctuations and Nma1 dosage. For visual clarity, Nma1 dosage is indicated at the bottom of each panel, and only Pho4 localization is depicted. Under normal Pi-replete (+Pi) conditions (left), Pho4 is cytoplasmic in WT, *NMA1*-oe, and *nma1∆* cells. During moderate Pi depletion (middle), Pho4 remains cytoplasmic in WT and *NMA1*-oe cells, whereas in *nma1Δ* cells, a portion of Pho4 translocate into the nucleus. In the absence of Pi (right), Pho4 is localized in the nucleus in WT and *nma1Δ* cells, whereas Pho4 is less concentrated in the nucleus in *NMA1*-oe cells. Nma1 modulates Pho4 localization likely by maintaining Pho4 in the cytoplasm. Shading indicates the relative abundance (fluorescence signal) of Pho4, shown as dark gray (low), light gray (intermediate), and white (high).

In this study, we investigate whether Nma1, a major NAD^+^ biosynthetic enzyme, has a direct role in modulating *PHO* signaling. Our results show that Nma1 physically interacts with Pho4 (Fig. 1B) and helps maintain Pho4 in the cytoplasm (Figs. 2A, 3B, and 4A), thereby downregulating *PHO* gene expression in Pi-depleted condition (Fig. 5). Overexpression of *NMA1* significantly diminishes Pi depletion-induced Pho4 nuclear localization (Fig. 2A). On the other hand, cells lacking Nma1 appear to be more sensitive to Pi depletion (Fig. 4A). These results support the model that Nma1 functions to maintain Pho4 in the cytoplasm (Fig. 6), which likely helps fine-tune *PHO* activation. Nma1 may help prevent hyperactivation of *PHO* signaling, which may otherwise increase NAD^+^ degradation by *PHO*-activated nucleotidases (Lee et al. 2026). In line with this, *PHO-*responsive genes are also repressed by the histone deacetylase Rpd3 (Vidal and Gaber 1991, Wongwisansri and Laybourn 2005, Korber and Barbaric 2014), and Rpd3 may help moderate the expression of *PHO*-responsive genes during NAD^+^ depletion to avoid excess NAD^+^ degradation (Groth et al. 2023). Our results also indicate that Nma1 is likely not required for the nuclear export and import of Pho4. Nuclear export and import of Pho4 are mediated by Msn5 and Pse1, respectively. Cells lacking Msn5 show a complete blockage of Pho4 nuclear export (Kaffman et al. 1998a). Similarly, cells lacking Pse1 fail to import Pho4 into the nucleus in response to Pi depletion (Kaffman et al. 1998b). Deleting *NMA1* does not block Pho4 nuclear import in Pi-depleted condition (0 mM) (Fig. 4A, left) nor does it block Pho4 nuclear export in high-Pi condition (1 mM) (Fig. 4A, right). These results support that Nma1 is not directly involved in the nuclear import and export of Pho4. However, it remains possible that Nma1 may affect the binding of Pho4 to the nuclear import and export receptors.

To understand the mechanism, we examined which functional domain of Nma1 is required to modulate Pho4 localization. Although Nma1 is a NAD^+^ biosynthetic enzyme, recent studies have suggested that Nma1 can also protect cells from proteotoxicity independent of its NAD^+^ synthesis activity (Pinkerton et al. 2021, Lee and Lin 2025). In line with this, NAD^+^ synthesis-deficient NMNATs in other organisms have been shown to reduce protein aggregates in neurodegenerative models, and the chaperone activity of NMNATs may contribute to the protection (Zhai et al. 2006, 2008, Ali et al. 2016). The N-terminal ATP-binding site (Saridakis and Pai 2003, Yalowitz et al. 2004, Zhai et al. 2006) and the C-terminal ATP-binding site (Zhai et al. 2008, Ali et al. 2016, Pinkerton et al. 2021) of NMNATs have been shown to confer NAD^+^ synthesis activity and chaperone activity, respectively. Of note, most of these studies were performed on the mammalian NMNATs. Our results show that the N-terminal ATP-binding site of Nma1 is dispensable for modulating Pho4 localization (Fig. 2A and B), indicating that the NAD^+^ synthesis activity (Fig. 2C) is not required for the modulation of Pho4 localization. Conversely, the C-terminal domain of Nma1, including the C-terminal ATP-binding site, is crucial in modulating Pho4 localization in response to Pi depletion (Fig. 3B and C). Moreover, the C-terminal domain of Nma1 is also required for the downregulation of *PHO*-responsive genes (Fig. 5B). These results suggest that the C-terminal domain of Nma1 is involved in the binding to Pho4. The chaperone-like ATPase activity of Nma1 may alter Pho4’s subcellular localization by inducing conformational changes and altering its interactions with other proteins. It is shown that the molecular chaperone Hsp90 modulates the activity of the transcription factor heat shock factor 1 (Hsf1) through two possible mechanisms. When complexed with Hsf1, Hsp90 may sequester Hsf1 monomers to prevent trimerization and translocation to the nucleus (Zou et al. 1998). It is also proposed that Hsp90 promotes the disassembly of Hsf1 trimers and the dissociation of Hsf1 from the heat shock element (Conde et al. 2009, Kijima et al. 2018). It is possible that Nma1 functions like Hsp90 to assist the binding or dissociation of Pho4 with other interacting proteins, which modulates Pho4 localization and transcriptional activity. In line with this, Nma1 localizes in the cytoplasm as well as in the nucleus (Fig. 4C). The cytoplasmic Nma1 may help maintain Pho4 in the cytoplasm by restraining Pho4 interaction with its nuclear import receptor Pse1. Similarly, the nuclear Nma1 may impede Pho4 binding to Pho2 or the DNA element to modulate the transcriptional activity of Pho4. Further studies on how Nma1 in the two subcellular compartments affects Pho4 should help elucidate the mechanisms. Additionally, it is shown that the human NMNAT2 functions as a co-chaperone to enhance the recognition of Hsp90 to pTau (Ali et al. 2016, Ma et al. 2020). Thus, it is also possible that Nma1 may function with other chaperones to modulate Pho4 localization and its transcriptional activity. It has been suggested that Hsp82 physically interacts with Nma1 (Truman et al. 2014), and therefore, Hsp82 may work with Nma1 to play a role in the regulation of Pho4 and *PHO* signaling.

On the other hand, Nma2, the paralog of Nma1, may also modulate *PHO* signaling because Nma1 and Nma2 share similar sequences, especially in the C-terminal region (Lee and Lin 2025). Supporting this, overexpressing *NMA2* downregulates the expression of the *PHO*-responsive gene *PHO5*; however, the effect is less significant compared to overexpressing *NMA1* (data not shown). These observations raise two possibilities. First, Nma2 may function like Nma1, which moderates *PHO* signaling by maintaining Pho4 in the cytoplasm. If this is the case, a physical interaction of Nma2 and Pho4 is expected. Moreover, deleting both Nma1 and Nma2 should lead to more drastic responses to moderate Pi depletion and *PHO* activation. However, deleting both Nma1 and Nma2 causes synthetic lethality in standard growth media. NR supplementation can rescue the lethal phenotype (Kato and Lin 2014), but it also makes cells totally dependent on NR salvage for survival, which may confound the study of *PHO* signaling (Lu and Lin 2011). Another possibility is that Nma2 may modulate *PHO* signaling via Nma1. It is reported that Nma1 and Nma2 function as tetramers to produce NAD^+^ (Natalini et al. 1986, Emanuelli et al. 1999, Emanuelli et al. 2003). Although Nma1 likely forms a homotetramer (Emanuelli et al. 1999), it may also form a heterotetramer with Nma2 (Krogan et al. 2006, Croft et al. 2018). Therefore, it is possible that Nma2 may not have a direct physical interaction with Pho4, and that overexpressing *NMA2* may modulate Pho4 localization or *PHO* signaling by forming functional tetramers with Nma1. Nevertheless, it remains unknown whether Nma1 and Nma2 need to form a tetramer to function as chaperones or whether the monomer could also perform chaperone activity equally well.

The Nma1-Pho4 interaction may provide an auxiliary regulatory mechanism for cells to modulate *PHO* signaling. However, it remains unresolved how Nma1 switches its role between a NAD^+^ biosynthetic enzyme and a molecular chaperone. Structural analysis reveals that the mouse NMNAT3 uses the positively charged enzymatic pocket to bind to the phosphorylated sites of pTau and the NAD^+^ synthesis substrates NMN and ATP (Ma et al. 2020). This finding suggests that pTau competes with NMN and ATP for the same binding site, which may help determine whether NMNATs function as NAD^+^ biosynthetic enzymes or molecular chaperones. It is possible that Pho4 also binds to the same substrate-binding site of Nma1. If Pho4 competes with NMN and ATP for binding to Nma1, increased Pho4-Nma1 binding is expected in NAD^+^ depletion and/or Pi depletion conditions because the concentrations of NMN and ATP are expected to be low. However, it would be difficult to address whether this indeed happens in the cell because Pi depletion also induces NAD^+^ degradation (Lee et al. 2026), which may increase NMN concentration. On the other hand, whether the phosphorylation status of Pho4 may affect its binding to Nma1 remains to be answered. It has been shown that Pho4 is phosphorylated at five sites, and some of these phosphorylated residues determine Pho4 binding to its interacting proteins. Phosphorylation of Pho4 at site 2 and site 3 is necessary to promote binding to its nuclear export receptor Msn5, whereas phosphorylation at site 4 is within the nuclear localization signal, which blocks Pho4 nuclear import by disrupting its binding to the nuclear import receptor Pse1 (Kaffman et al. 1998b, Komeili and O'Shea 1999, Springer et al. 2003). Additionally, phosphorylation of Pho4 at site 5 modulates its binding to Pho2 (Komeili and O'Shea 1999). The function of the phosphorylation of Pho4 at site 1 remains unclear. Nevertheless, our data suggest that Pho4 phosphorylation may not be required for binding to Nma1 because Nma1-Pho4 interaction was not altered by Pi availability (Fig. 1B).

Lastly, the Nma1-Pho4 interaction may help coordinate NAD^+^ metabolism and Pi homeostasis. Previously, we showed that the protein levels of all three yeast NMNATs, including Nma1, are downregulated by Pi depletion (Lee et al. 2026). When Pi is limited, NAD^+^ synthesis is likely decelerated due to the downregulation of the rate-limiting NAD^+^ biosynthetic enzyme Nma1 and the reduced ATP levels. In line with this, it is shown that cellular NAD^+^ levels are decreased by both Pi depletion (Groth et al. 2023) and ATP limitation (Pinson et al. 2019). In this study, we show that Nma1 attenuates the activation of *PHO* signaling (Fig. 5). The downregulation of Nma1 protein levels in response to Pi depletion may help co-regulate *PHO* signaling and the NAD^+^ biosynthesis pathways. For example, in Pi-depleted conditions, reduced Nma1 expression inevitably decreases NAD^+^ synthesis, and it also promotes *PHO* activation because Pho4 is enriched in the nucleus. Supporting this, we show that cells lacking Nma1 are more sensitive to Pi depletion (Fig. 4A and B). Interestingly, *PHO* activation modulates NAD^+^ metabolism by enhancing NR salvage activity (Lu and Lin 2011). The two *PHO*-responsive genes examined in this study, Pho5 and Phm8, have been shown to degrade NMN to NR (Lu et al. 2009, Lee et al. 2026). Moreover, Phm8 also plays a role in nucleotide metabolism by degrading nucleoside monophosphate into nucleoside (Xu et al. 2013). These two processes release the Pi group from NAD^+^ and nucleotide, respectively, suggesting that NAD^+^ metabolism and nucleotide metabolism also play a role in Pi homeostasis. It has been suggested that NAD^+^ and nucleotides are putative Pi “sinks” (Gupta and Laxman 2021) and, therefore, cells may adapt to Pi limitation by coordinating *PHO* signaling, NAD^+^ metabolism, and nucleotide metabolism. Overall, our results support that Nma1 is a dual-function enzyme that co-regulates NAD^+^ metabolism and *PHO* signaling. We also uncover a non-canonical role for Nma1 in tuning the magnitude of *PHO* activation.

### Conclusions and perspectives

Modulating NAD^+^ metabolism is an emerging therapeutic approach for many human diseases (Katsyuba et al. 2020, Xie et al. 2020, Alghamdi and Braidy 2024, Lautrup et al. 2024, Migaud et al. 2024). Due to the involvement of NMNATs in all NAD^+^ biosynthetic pathways, targeting NMNATs appears to be an efficient route to modulate NAD^+^ levels. Moreover, the multifaceted roles of NMNATs may help ameliorate human diseases independent of the NAD^+^ effect. For example, enhancing the expression level and activity of NMNATs could be beneficial in the treatment of neurodegenerative diseases due to their NAD^+^ synthesis activity and/or chaperone activity, depending on the context (Lee and Lin 2025). To date, the regulation of NAD^+^ metabolism is still incompletely understood, owing to the interplay between NAD^+^ homeostasis and other signaling networks. Intracellular compartmentalization of NAD^+^ metabolites and the factors involved in the co-regulation of NAD^+^ and nutrient homeostasis also add complexity to the understanding of the mechanisms. Nevertheless, simple model systems such as budding yeast may serve as efficient tools for studying the mechanistic aspects of the co-regulation of NAD^+^ metabolism and nutrient-sensing pathways. Our studies have revealed a novel role for a yeast NMNAT, Nma1, in the crosstalk between the regulation of NAD^+^ metabolism and phosphate homeostasis. This study highlights a moonlighting chaperone-like function of Nma1 in the regulation of a nutrient-sensing pathway. Although Nma1 appears to mediate this regulation independent of its NAD^+^ synthesis activity, it is important to note that proper cell function depends on maintaining optimal NAD^+^ levels and that NMNATs can modulate cellular processes primarily by supporting NAD^+^ homeostasis. Understanding the crosstalk between NAD^+^ and other pathways and their integrative impacts will ultimately provide important insights into the metabolic homeostasis crucial for cellular integrity.

## Data Availability

All data are contained within this article.
